# Anakinra-Dependent Recurrent Pericarditis: The Role of the R202Q Variant of the MEFV Gene

**DOI:** 10.3390/jcm13206051

**Published:** 2024-10-11

**Authors:** Alessandro Andreis, Federica Currò Dossi, Gaetano Maria De Ferrari, Gianluca Alunni, Massimo Imazio

**Affiliations:** 1 Advanced Cardiovascular Echocardiography Unit, Cardiovascular and Thoracic Department, Città della Salute e della Scienza di Torino University Hospital, 10126 Turin, Italy; 2 Division of Cardiology, Città della Salute e della Scienza di Torino University Hospital, 10126 Turin, Italy; 3 Department of Medical Sciences, University of Torino, 10126 Turin, Italy; 4 Department of Medicine (DMED), University of Udine, 33100 Udine, Italy; 5 Cardiothoracic Department, University Hospital Santa Maria della Misericordia, 33100 Udine, Italy

**Keywords:** recurrent pericarditis, MEFV, R202Q, anti-IL-1, anakinra

## Abstract

**Background**: the role of the R202Q (c.605G>A, p.Arg202Gln) missense variant of the MEFV gene has been debated as either a benign polymorphism or a potentially pathogenic mutation. We report and discuss here the case of a young female with corticosteroid-dependent recurrent pericarditis carrying the homozygous R202Q variant, exhibiting distinctive clinical features possibly influenced by this genetic variant. **Methods**: a 30-year-old woman with a previous diagnosis of cancer and recent respiratory infection presented with severe pleuritic chest pain, hypotension, tachycardia, and fever. Initial diagnostic evaluation indicated cardiac tamponade, and emergent pericardiocentesis was performed. Despite initial treatment with NSAIDs, colchicine, and corticosteroids, the patient experienced multiple recurrences. Genetic testing identified homozygous R202Q variant in the MEFV gene. Given the corticosteroid dependency and recurrent nature of her condition, IL-1 inhibitor anakinra was introduced, leading to significant improvement, although tapering below 150 mg per week failed to prevent recurrences. **Results**: the introduction of anakinra resulted in rapid symptom relief and resolution of pericardial effusion. However, attempts to taper or discontinue anakinra led to pericarditis recurrences. Ultimately, a maintenance dose of 50 mg every three days was established, which maintained remission for 18 months without recurrence. Despite multiple tapering attempts, further reduction in anakinra dosage was unsuccessful without triggering relapses. **Conclusions**: the R202Q variant, although typically considered benign, may contribute to an autoinflammatory phenotype resembling familial Mediterranean fever. This case underscores the potential pathogenicity of the homozygous R202Q variant in recurrent pericarditis and its responsiveness to IL-1 inhibition. In patients with corticosteroid-dependent recurrent pericarditis, genetic testing for the R202Q variant should be considered when anti-IL-1 drugs cannot be withdrawn. Further studies are warranted to elucidate the variant’s role in pericardial inflammation and guide personalized treatment strategies.

## 1. Introduction

Recurrent pericarditis presents a substantial clinical challenge for physicians, frequently marked by the diminishing clarity of classical diagnostic criteria over time and a possible gradual reduction in the effectiveness of conventional anti-inflammatory treatments [1,2]. The management of recurrent pericarditis, especially in cases that become corticosteroid dependent, remains complex. Among the various genetic factors potentially implicated in recurrent pericarditis, the R202Q (c.605G>A, p.Arg202Gln) missense variant in the MEFV gene is particularly controversial since it is currently considered as a benign polymorphism rather than a pathogenic mutation [3,4]. Genetic variants in MEFV gene, primarily associated with familial Mediterranean fever (FMF), have been linked to various autoinflammatory conditions, including pericarditis. This study presents the case of a young woman with corticosteroid-dependent recurrent pericarditis who is homozygous for the R202Q variant. Through this case, we aim to explore the potential pathogenic role of this genetic variant and the effectiveness of targeted treatment with the IL-1 inhibitor anakinra.

## 2. Case Presentation

### 2.1. Initial Presentation and Medical History

A 30-year-old woman was admitted to the Emergency Department complaining of worsening pleuritic chest pain and dyspnea. She appeared pale and confused, with vital signs indicating hypotension (BP 70/40 mmHg), tachycardia, and fever. Physical examination revealed muffled heart sounds and jugular distention, suggesting possible cardiac involvement.

Her past medical history included cervical adenocarcinoma (stage FIGO-IB1-G3) treated with surgery and chemotherapy (carboplatin and paclitaxel) one year prior to admission. Five months before her current assessment, the patient experienced an upper respiratory tract infection that was rapidly followed by onset of intense pleuritic chest pain radiating to the interscapular region. This pain was exacerbated by lying down and was minimally relieved by paracetamol. Her symptoms resolved spontaneously after about 20 days. However, a few weeks later, she presented again to the hospital with recurrent pleuritic chest pain, fever, and rhinorrhea. A clinical diagnosis of pericarditis was made, and she was prescribed ibuprofen and antibiotics.

### 2.2. Current Assessment and Differential Diagnosis

Upon the current assessment, initial differential diagnoses included cardiac tamponade, recurrent pericarditis, myocarditis, myocardial infarction, spontaneous coronary artery dissection, and pulmonary embolism. Electrocardiogram (ECG) showed sinus tachycardia, low QRS voltages, and subtle diffuse ST elevation with PR depression. Transthoracic echocardiogram (TTE) revealed moderate pericardial effusion associated with right chambers diastolic collapse, 46% respiratory variation across mitral valve, and plethoric inferior vena cava. Laboratory results revealed elevated levels of C-reactive protein (CRP) (241 mg/L) and white blood cell (WBC) count (13.39 × 10^9^/L). Troponin T (Tn T) levels were normal (high sensitivity TnT < 3 ng/L).

Emergent pericardiocentesis was performed because of cardiac tamponade, with drainage of 150 mL of serous fluid and promptly stabilizing the patient’s hemodynamics. Pericardial fluid cytology was negative for malignancy and bacterial infections, and biochemical analysis was consistent with an exudate. An autoimmune panel was conducted to investigate possible underlying autoimmune diseases, but results for anti-double-stranded DNA, antinuclear, antineutrophilic cytoplasmic, extractable nuclear antigen, and anticytoplasmic antibodies were all negative. Complement levels (C3 and C4) were normal. Assessment of tumor markers revealed increased levels of cancer antigen 125 (63 UI/mL). A multidisciplinary consultation with oncologists and gynecologists followed, leading to cervical biopsy and chest, abdomen, and pelvis CT scans, which showed no evidence of cancer recurrence. Given the time that had elapsed since cancer treatment and the onset of pericarditis, along with the available clinical data, a direct causal relationship was deemed unlikely. Thus, the diagnosis of recurrent pericarditis of idiopathic etiology, presenting with cardiac tamponade, was confirmed.

### 2.3. Patient Management

**Figure 1:**
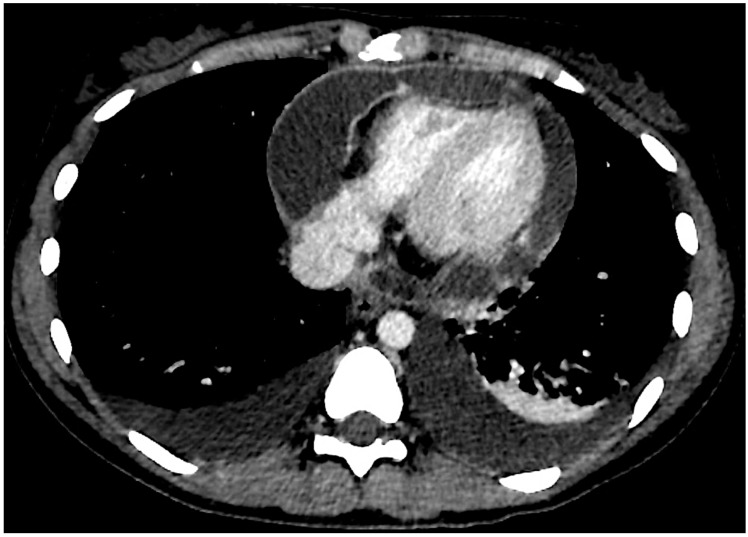
Axial chest CT scan section showing pleural and pericardial effusion. Pericardial enhancement is visible.

The patient was initially treated with ibuprofen (600 mg, administered three times daily) and colchicine (0.5 mg once daily), resulting in positive clinical response, resolution of chest pain, improvement in TTE findings (trace pericardial effusion), normalization of ECG, and CRP (0.4 mg/dL). However, one and a half months later, she presented again to the Emergency Department complaining of severe recurrent pleuritic chest pain associated with fever and vomiting. She was still on colchicine treatment while ibuprofen was gradually tapered by 400 mg per week until it was discontinued. She showed severe pericardial effusion with partial signs of hemodynamic compromise, while laboratory tests showed increased CRP (299 mg/L) and WBC (20.63 × 10^9^/L). A chest CT scan was performed (Figure 1), showing polyserositis along with pericardial enhancement. The patient was then admitted for recurrent pericarditis and treated with elective pericardiocentesis, indomethacin (25 mg three times per day), prednisone (25 mg, 0.46 mg/kg), and colchicine (0.5 mg per day), leading to a good clinical response and resolution of pericardial effusion. However, TTE revealed signs of pericardial constriction (septal bounce, anulus reversus, and anulus paradoxus).

**Figure 2:**
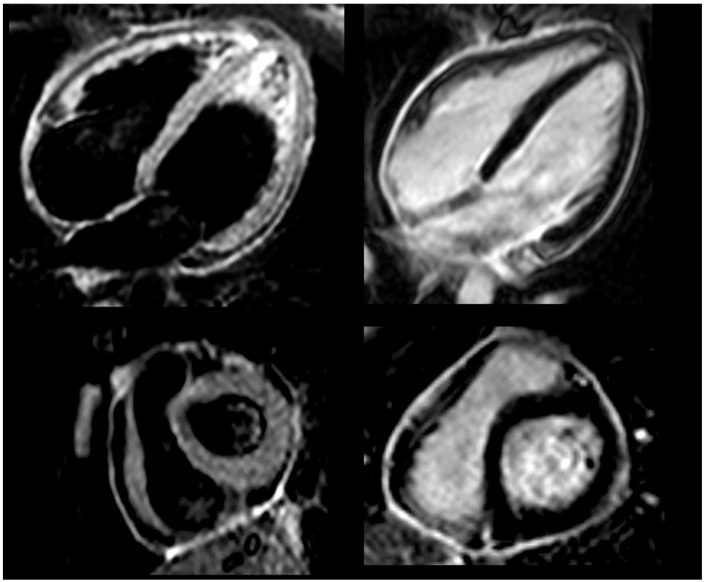
Cardiac magnetic resonance before anti-IL-1 treatment, showing bright signal in T2-weighted black blood STIR sequences (**left** panel) and intense delayed pericardial enhancement (**right** panel).

Three months later, during gradual prednisone tapering (actual dose of 12.5 mg daily), the patient was admitted again because of a pericarditis recurrence (4th flare within seven months), presenting with chest pain and fever. Laboratory tests showed increased CRP (29 mg/L). Efforts to manage symptoms by escalating nonsteroidal drugs (increasing indomethacin to 50 mg three times daily) proved ineffective, as significant reduction in chest pain or normalization of CRP levels was not achieved. Cardiac MRI (Figure 2) indicated ongoing pericardial inflammation but normal biventricular size, systolic function, and no myocardial involvement. Septal bounce and mild pericardial thickening were observed. Clinical data were consistent with idiopathic corticosteroid-dependent recurrent pericarditis with inflammatory phenotype and signs of pericardial constriction. Genetic testing for the most common genetic variants associated with recurrent pericarditis identified a homozygous c.605G>A, p. Arg202 Gln (R202Q) variant in the MEFV gene. In addition to MEFV, other genes (e.g., NLRP3 and TNFRSF1A) were also screened, and no additional genetic variants were found.

Following targeted laboratory screening tests (quantiferon TB, HCV, HBV, and HIV), anti-IL1 treatment with anakinra was initiated. The protocol involved administering 100 mg via subcutaneous injection once daily for six months, followed by 100 mg every alternate day for the next three months, and subsequently 50 mg every other day for an additional three months. Concurrently, indomethacin was rapidly withdrawn, while colchicine was maintained, and prednisone tapered over a month. The patient experienced rapid relief from chest pain within a few days. One month later, transthoracic echocardiogram showed resolution of pericardial constriction. After discharge, she underwent outpatient assessments for the next 12 months, exhibiting no evidence of pericarditis. Anakinra was then discontinued.

**Figure 3:**
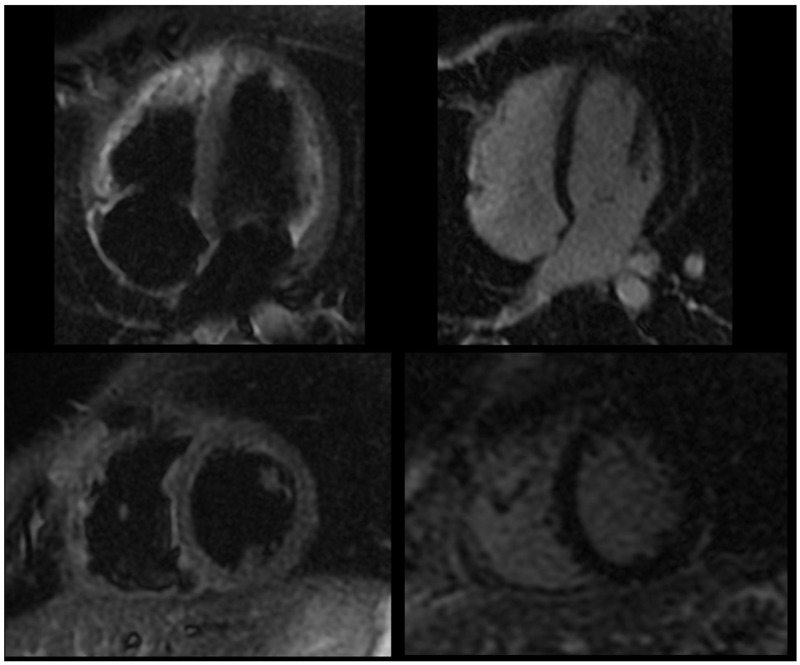
Follow-up cardiac magnetic resonance following anti-IL-1 treatment, showing dark signal in T2-weighted black blood STIR sequences (**left** panel) and no significant delayed pericardial enhancement (**right** panel).

Less than a week after stopping anakinra, the patient had pharyngotonsillitis followed by recurrence of pleuritic chest pain. A second cycle of anakinra was then initiated, providing complete clinical benefit. The treatment schedule involved 100 mg daily for six months, transitioning to 100 mg every other day for the subsequent three months, and then 50 mg every other day for an additional three months. Over a year later, a recurrence of pericarditis occurred following the discontinuation of anakinra, without any evident infectious cause. Consequently, a third cycle was initiated with a revised tapering plan: anakinra was administered at 100 mg daily for six months, then reduced to 100 mg every other day for three months, followed by 50 mg every other day for another three months, and finally 50 mg every three days for a further three months. Follow up cardiac MRI did not show signs of residual pericardial inflammation (Figure 3).

Despite plans to discontinue anakinra, a new episode of pericarditis occurred during the final tapering phase (50 mg every three days), following the occurrence of a dental abscess. A fourth cycle started with 100 mg daily for one week, then 50 mg every other day for three months, followed by 50 mg every other day. Multiple further unsuccessful attempts of tapering below this weekly dose were tried in the following months, without possibility of reducing anakinra under the minimum threshold of 50 mg three days a week. However, with this dose (150 mg per week) the patient has been free from pericarditis recurrences for the following 18 months, as observed through periodic (quarterly) outpatient visits. The patient is still well under this treatment, asymptomatic, with normal CRP and WBC, and no pericardial effusion on serial transthoracic echocardiogram assessment. Written and oral informed consent was obtained from the patient in accordance with all applicable ethical standards for the use of the clinical data reported.

## 3. Discussion

### 3.1. Diagnostic Challenges in Pericardial Diseases

Acute pericarditis, resulting from a local or systemic inflammatory trigger involving the pericardium, is the most common among pericardial syndromes [5]. Indeed, pericarditis represents 5% of Emergency Department visits for chest pain [6]. According to the guidelines issued by the European Society of Cardiology, the diagnosis is made when two or more criteria are present: (1) pleuritic chest pain, (2) friction rubs on physical examination, (3) abnormal ECG (widespread ST segment elevation or PR segment depression), or (4) new or worsening pericardial effusion. Inflammatory indicators, including CRP, erythrocyte sedimentation rate, and white blood cell count, as well as the identification of pericardial inflammation through imaging methods like cardiac magnetic resonance or computed tomography, are regarded as supplementary evidence. Furthermore, these imaging tests may be useful to monitor the grade of inflammation and the effect of treatment [5,7]. The diagnosis of pericarditis may be challenging in daily practice, since not all patients meet the guideline’s recommended criteria, especially those with multiple recurrences. Newer diagnostic tools have been developed for this purpose, such as low-grade inflammation score (INFLA-score), a useful tool to assess the risk of pericarditis, with high diagnostic accuracy, even in patients with normal CRP [8].

In developed nations, the primary causes of pericarditis are often viral or idiopathic, whereas in low-income countries, tuberculosis is the predominant cause. A careful diagnostic work-up should always be performed in clinical practice, considering that neoplastic and bacterial etiology have both been associated with a complicated course of pericarditis, due to increased risk of incessant pericarditis or effusion causing cardiac tamponade [9]. Indeed, in the case described here, a complete diagnostic work-up was performed, including an autoimmune panel, pericardial fluid analysis, and chest, abdomen, and pelvis CT scan.

### 3.2. Management of Acute and Recurrent Pericardial Inflammation

Aspirin and non-steroidal anti-inflammatory drugs (NSAIDs) are the first-line treatment for pericarditis, effective to control symptoms and reduce recurrences, although these drugs have never been assessed in large RCTs5. Ibuprofen and indomethacin proved effective to resolve chest pain symptoms within 24 h in a study on post pericardial syndrome pericarditis [10].

In the management of acute and recurrent pericarditis, colchicine is now recommended as a first-line treatment alongside aspirin and NSAIDs. Colchicine is an ancient medication that was predominantly used in previous centuries as an anti-inflammatory treatment for acute gout and familial Mediterranean fever [11]. It is a lipophilic tricyclic alkaloid with a 44% bioavailability after oral ingestion, reaching peak serum concentrations in 2 h. Its metabolism is mainly hepatic through CYP3A4, followed by intestinal and urinary excretion [12]. Efficacious blood concentrations range between 0.5 and 3 ng/mL, and adverse effects have been observed with concentrations over 3 ng/mL [13]. In fact, gastro-intestinal symptoms, mainly diarrhea, are the most frequent side effect during colchicine treatment (RR = 1.67). A possible reason for the increased vulnerability of intestinal epithelium may be due to its high turnover rate [14]. However, a lower dosage of colchicine (0.5 mg per day) was not associated with gastrointestinal events, as reported in a large metanalysis [15]. Rarer side effects include transaminases elevation (<5%), leukopenia, and alopecia (<0.1%). Opposite to corticosteroids or nonsteroidal anti-inflammatory drugs, long-term treatment with colchicine is possible without additional risks [16,17]. Colchicine reaches high concentrations in erythrocytes and neutrophils, promoting microtubules depolymerization with subsequent impairment of vesicular trafficking, preventing macrophages activation [18]. A second effect of colchicine is the inhibition of caspase 1, which is responsible for producing active inflammatory cytokines interleukin-1 beta (IL-1β) and IL-18 leading to NOD-like receptor pyrin containing domain 3 (NLRP3) inflammasome activation, boosting the downstream inflammatory cascade. The COlchicine for acute Pericarditis (COPE) trial was the first study to demonstrate the efficacy of colchicine for the reduction in both recurrences (11% in patients receiving colchicine vs. 32% other patients, *p* < 0.01) and symptoms (respectively, 12% vs. 37% chest pain at 72 h, *p* < 0.01), when given for three months in patients with acute pericarditis. The subsequent Investigation on Colchicine for Acute Pericarditis (ICAP) study confirmed these findings, while the role of colchicine for the prevention of further recurrences was demonstrated in the COlchicine for REcurrent pericarditis (CORE) and Colchicine for Recurrent Pericarditis (CORP) studies, in which patients received colchicine for 6 months, with a significant benefit on the subsequent incidence of further recurrences, respectively, 24% vs. 51% (*p* = 0.02) and 24% vs. 55% (*p* < 0.01).

Corticosteroids, despite a rapid onset of action and strong efficacy in the inhibition of pericardial inflammation, can lead to systemic side effects and heighten the risk of recurrences, particularly when given in high doses (such as 1–1.5 mg/kg per day) and tapered off quickly [19]. Corticosteroids should be avoided if there is a suspicion of bacterial infection or tuberculosis. This treatment, which is usually not advised for acute pericarditis, should be reserved for patients with particular conditions, such as systemic inflammatory diseases, post-pericardiotomy syndromes, pregnancy, or those who cannot tolerate NSAIDs, such as those with allergies, peptic ulcers, gastrointestinal bleeding, or a high bleeding risk due to oral anticoagulant therapy, as well as those with persistent symptoms despite appropriate treatment. Lower dosages (e.g., prednisone 0.2 to 0.5 mg/kg/day) are usually recommended in these cases, with the goal of achieving better symptom control.

Corticosteroid tapering should commence once C-reactive protein levels have normalized and symptoms have improved, with reductions made very gradually, such as 1.0 to 2.5 mg every 2 to 6 weeks [20]. Clinicians must be vigilant for corticosteroid-related side effects, which can include a heightened risk of infections, secondary hypertension, insulin resistance, osteoporosis, insomnia, and psychosis. For patients undergoing extended steroid therapy, it is advisable to prevent osteoporosis through calcium and vitamin D supplementation: total calcium intake (from supplements and diet combined) should be between 1200 and 1500 mg per day, and vitamin D supplementation should range from 800 to 1000 IU per day. Moreover, bisphosphonates are recommended to prevent bone loss in men aged 50 and older and postmenopausal women who are on long-term glucocorticoid therapy at doses of 5.0–7.5 mg per day of prednisone or an equivalent glucocorticoid.

Clinical course of pericarditis may be acute, recurrent, incessant, or chronic. Recurrent pericarditis is the most frequent complication following the first episode, occurring in 15 to 30% patients, and is defined as chest pain relapse with evidence of pericardial inflammation after a minimum symptom free period of 4–6 weeks. The likelihood of recurrence can rise to 50% in patients with multiple prior episodes, those who have not been treated with colchicine [1,2,7,21,22], or those who received high doses of corticosteroids during the initial episode of pericardial inflammation. Inadequate management of the first episode is often a significant factor contributing to recurrence [3]. Although recurrent pericarditis typically has a favorable prognosis, it can adversely affect the patient’s quality of life and continues to pose a considerable challenge for clinicians, particularly when the patient becomes resistant to standard treatments [23]. Indeed, some patients (about 5%) may complain of persistent symptoms, despite receiving a combination of aspirin or NSAIDs, colchicine, and corticosteroids, commonly worsening when the corticosteroid dose is reduced under a critical threshold, defined as corticosteroid-dependent and colchicine-resistant recurrent pericarditis [24]. Following the initial episode of pericardial inflammation, a chronic low-grade inflammation may continue, particularly in patients with a predisposition to auto-reactive processes. In such cases, recurrent symptoms can be triggered by a wide range of infectious or non-infectious factors that reactivate the inflammatory cascade. Although the precise mechanisms behind this phenomenon are not yet fully understood, it is hypothesized that patients with a typical “inflammatory phenotype” (characterized by fever and elevated C-reactive protein) may experience an autoinflammatory response driven by interleukin-1 (IL-1) overproduction. Conversely, in patients with concurrent autoimmune systemic disease who exhibit a non-inflammatory phenotype, an autoimmune response involving the type I interferon pathway might be more prominent [25,26,27]. For patients with recurrent pericarditis presenting with an inflammatory phenotype, a treatment strategy focused on IL-1 inhibition would be appropriate. In those with a non-inflammatory phenotype, if an autoimmune disease is identified, intravenous immunoglobulin (IvIG) may be beneficial. If no autoimmune disease is recognized, azathioprine might be considered as a treatment option.

The newer anti-IL-1 agents have proven highly effective in treating recurrent pericarditis, particularly in cases that are corticosteroid-dependent and colchicine-resistant [28]. The main anti-IL-1 agents available are anakinra, canakinumab, and rilonacept [5]. Anakinra inhibits both IL-1α and IL-1β, and canakinumab is a human monoclonal antibody targeting IL-1β, while rilonacept functions as an IL-1 trap cytokine inhibitor. Among these, only anakinra and rilonacept have been assessed in randomized controlled trials. All these agents are administered via subcutaneous injection [29].

Anakinra is a recombinant, non-glycosylated human IL-1 receptor antagonist that blocks both IL-1α and IL-1β. It has a half-life of 4–6 h and is administered daily. The most frequent adverse effect is injection-site reactions, which typically occur within the first week of treatment and generally resolve within two months [30]. Anakinra is cleared by the kidneys, so dose adjustments are required for patients with severe renal impairment or end-stage renal disease. The main contraindication is hypersensitivity to E. coli-derived proteins or any component of the medication [30]. Prior to initiating treatment, it is important to screen for latent tuberculosis due to the increased risk of reactivation [31]. The efficacy of anakinra was established through the AIRTRIP trial, a double-blind, placebo-controlled, randomized withdrawal study involving 21 patients with recurrent pericarditis with colchicine resistance and corticosteroid dependence. Anakinra was administered at a dosage of 2 mg/kg daily (up to 100 mg) for 2 months. Patients who responded to treatment were then randomized to either continue anakinra or switch to a placebo for 6 months, or until the recurrence of pericarditis. The study demonstrated that anakinra notably reduced relapse rates, with local skin reactions being the most frequent adverse effect (95%), followed by transient increases in transaminases (14.3%). The findings highlighted the importance of patient selection, suggesting that those with an inflammatory phenotype (such as fever and elevated inflammatory markers) are more likely to benefit from anakinra therapy. A very gradual tapering schedule is recommended following a minimum of 6 months of full treatment. Over a median follow-up period of 14 months, recurrence of pericarditis occurred in 18% of patients on anakinra, compared to 90% in those receiving a placebo. Subsequent observational studies in larger real-world cohorts have reinforced these results and indicated a potential role for anakinra in preventing constrictive pericarditis [28,32]. Canakinumab is a human monoclonal antibody targeting IL-1β with a half-life of 22–26 days, typically administered at 150 mg per month in adults. Its application in pericarditis is constrained by high costs and limited supporting evidence, mostly from case reports and small series. Canakinumab might be considered for patients who are unable to tolerate anakinra [33,34]. Rilonacept, a recombinant fusion protein administered weekly via subcutaneous injection, non-selectively inhibits IL-1α and IL-1β signaling. It is approved for the treatment of Cryopyrin-Associated Periodic Syndromes (CAPSs) and has also received FDA approval for recurrent pericarditis. In a phase-3 RCT, rilonacept significantly reduced the risk of pericarditis recurrence, with patients showing rapid symptom resolution and normalization of CRP levels. The most frequent adverse events included injection-site reactions (34%) and respiratory infections of the upper tract (22%) [35].

### 3.3. The Role of Genetic Factors

Genetic polymorphisms may contribute to some idiopathic recurrent pericarditis cases. Commonly reported genes include MEFV (Mediterranean fever gene), NLRP3 (nod like receptor pyrin domain containing 3), and TNFRSF1A (tumor necrosis factor receptor superfamily 1A), each with a unique role in the inflammatory process [36,37].

In the patient presented here, genetic testing revealed a homozygous c.605G>A, p.Arg202Gln (R202Q) variant in the MEFV gene, located on chromosome 16, exon 2. The role of this missense variant is currently considered a benign polymorphism rather than a pathogenic mutation [3,4,38].

Mutations in the MEFV gene can lead to familial Mediterranean fever (FMF), an autoinflammatory disease, typically inherited as an autosomal recessive disorder, characterized by recurrent fevers and serositis. FMF diagnosis is confirmed by identifying mutations in the MEFV gene, which encodes a 781-amino acid protein called pyrin—named after the Greek word for fever—or “Marenostrin”, referencing the Mediterranean Sea, due to the high prevalence of FMF in populations such as Armenians, Non Ashkenazi Jews, Turkish, and Arabs. FMF is characterized by recurrent episodes of fever and serositis, with peritonitis being the most common initial manifestation (93%), followed by arthritis, pleuritis, pericarditis, myalgias, and erysipelas-like erythema. It is also associated with elevated acute phase reactants, followed by clinical and biochemical remission [39]. The incidence of pericarditis in FMF is around 0.7–1.4%, but in some rare cases, it can be the main manifestation, even in recurrent types [40,41,42]. Diagnosis is based on Tel-Hashomer clinical criteria or those simplified by Livneh et al. and is confirmed by identifying two pathogenic mutations in the MEFV gene [43].

The R202Q variant has typically been considered a benign polymorphism, unlike higher-penetrance mutations such as M694V and V726A in the MEFV gene, which are associated with more severe forms of familial Mediterranean fever (FMF). However, recent evidence from case reports and small cohort studies suggests that homozygous R202Q may be linked to milder autoinflammatory conditions, including recurrent pericarditis. Epidemiological data indicate that this variant is more frequently found in Mediterranean populations, particularly among those with a history of FMF. Several mutations are strongly associated with the FMF phenotype, with the most common being V726A, M694V, M694I, M680I (located on exon 10), and E148Q (exon 2). Among these, the M694V mutation is noted for its high penetrance and is often associated with severe clinical presentations, such as frequent chest pain, high fever, splenomegaly, erysipelas, arthritis, and myalgias. The c.605G>A, p.Arg202Gln (R202Q) MEFV variant, located on exon 2, is a common polymorphism that frequently occurs in linkage disequilibrium with the M694V mutation. While the heterozygous form of this variant is not linked to autoinflammatory diseases, the homozygous variant is rare but has been associated with “FMF-like” disorders, especially when combined with other mutations [3,44,45]. In patients with FMF, the presence of the R202Q homozygous genetic variant is often linked to milder and atypical symptoms, potentially due to incomplete penetrance or variable expressivity. These patients generally respond well to colchicine treatment. For instance, Karaman et al. reported a case of a 27-year-old Turkish man with recurrent pericarditis, who was found to have a compound heterozygous genetic variant involving R202Q and M694V [46].

In addition, recent studies using mouse models and cellular assays demonstrate that the R202Q variant can promote IL-1 beta secretion through activation of the NLRP3 inflammasome, similar to other MEFV genetic variants. this suggests that, while R202Q is often considered benign, it may function as a modifier gene, causing an autoinflammatory response under specific conditions. Such studies have been crucial in understanding the role of this variant in autoinflammatory diseases [47,48,49].

### 3.4. Treatment Strategies and Long-Term Management

Despite lacking a definite FMF diagnosis and carrying a single genetic variant not considered pathogenic, the case of the young woman with recurrent pericarditis presented here is quite emblematic. Pyrin regulates inflammasome activity in innate immunity cells, expressed in neutrophils, monocytes, and dendritic cells. The functional N-terminal pyrin domain interacts with the adaptor protein ASC (apoptosis-associated speck like protein), contributing to inflammasome complex assembly through caspase-1 activation and the Caspase Recruitment domain, leading to the activation and secretion of IL-1β. MEFV variants that result in a gain of function cause caspase-1 activation and subsequent IL-1 inflammatory cascade. This pathway may explain colchicine’s efficacy in treating FMF and recurrent pericarditis, as it inhibits the self-assembly of cytoskeleton microtubules and reduces NLRP3-dependent IL-1β release [47,50]. Similarly, the recombinant IL-1 receptor antagonist anakinra has proven safe and effective in treating both FMF and corticosteroid-dependent recurrent pericarditis [28].

In the context of recurrent pericarditis, evidence regarding the optimal tapering protocol is still limited. Indeed, a longer tapering protocol and an earlier initiation of anti-IL-1 drug were both associated with lower risk of recurrence [28,51]. The challenges encountered during attempts to taper anakinra underscore the need for individualized approaches to dose adjustment. Continuous monitoring and patient-specific tapering protocols are crucial to maintaining long-term remission and preventing recurrence.

## 4. Conclusions

In the patient presented here, several tapering protocols were attempted to completely discontinue anti-IL-1 treatment, but all were unsuccessful. Ultimately, we achieved acceptable control of pericardial inflammation by reducing the weekly dose of the anti-IL-1 drug to the lowest possible level. In our opinion, this represents the minimum dose necessary to manage inflammasome hyperactivation in this patient, whose pathophysiology is consistent with the R202Q missense variant.

We hypothesize that the R202Q missense variant may have a pathogenic role in this patient. This case highlights the need for personalized treatment strategies, and we recommend genetic testing for the R202Q variant in patients with recurrent pericarditis who fail to withdraw from anti-IL-1 therapies; indeed, the therapeutic response observed in this case may not be generalizable to all patients with this variant. However, it does suggest that in homozygous cases, R202Q may act as a disease modifier that contributes to the autoinflammatory phenotype. Further studies are necessary to confirm this and guide personalized treatment strategies.

## Data Availability

The original contributions presented in this study are included in this article; further inquiries can be directed to the corresponding author.
